# Twitching motility of *Stenotrophomonas maltophilia* under iron limitation: *In-silico*, phenotypic and proteomic approaches

**DOI:** 10.1080/21505594.2020.1713649

**Published:** 2020-01-20

**Authors:** Kalidasan V., Vasantha Kumari Neela

**Affiliations:** Department of Medical Microbiology and Parasitology, Faculty of Medicine and Health Sciences, Universiti Putra Malaysia, Serdang, Malaysia

**Keywords:** *Stenotrophomonas maltophilia*, iron depletion, RAST server, twitching motility, ITRAQ, type IV pili

## Abstract

This study investigates the twitching ability of 28 clinical and five environmental strains of *S. maltophilia* grown under iron-depleted condition through *in-silico*, phenotypic and proteomics approaches. Rapid Annotations using Subsystem Technology (RAST) analysis revealed the presence of 21 targets of type IV pilus shared across *S. maltophilia* strains K279a, R551-3, D457 and JV3. The macroscopic twitching assay showed that only clinical isolates produced a zone of twitching with a mean of 22.00 mm under normal and 25.00 mm under iron-depleted conditions. (p = 0.002). Environmental isolates did not show any significant twitching activity in both conditions tested. Isobaric Tags for Relative and Absolute Quantification (ITRAQ) analysis showed altered expression of twitching motility protein PilT (99.08-fold change), flagellar biosynthesis protein FliC (20.14-fold change), and fimbrial protein (0.70-fold change) in response to iron-depleted condition. Most of the strains that have the ability to twitch under the normal condition, exhibit enhanced twitching during iron limitation.

## Introduction

The prevalence of nosocomial infections caused by a Gram-negative opportunistic pathogen, *Stenotrophomonas maltophilia*, has remarkably increased in recent years [1–7]. *S. maltophilia* is ubiquitously widespread in the water, soil, plants, animals, and foods [8,9]. In hospital settings, the pathogen has been isolated from various medical devices, disinfectant solutions, and found as part of endogenous flora of healthcare workers [10–13]. These serve as the source of transmission among patient who is immunocompromised, with indwelling medical devices, admitted to intensive care unit (ICU), on broad-spectrum antibiotics, prolonged hospitalization and exposed mucocutaneous barrier. The potential transmission of *S. maltophilia* is mostly contributed by the intrinsic resistance to multiple antibiotics [14–17] and the presence of virulence factors [18–21] in establishing its pathogenesis.

In general, pathogens express their virulence factors such as exotoxins, extracellular enzymes, biofilm formation, quorum sensing, etc. in order to access new niches [22]. In such circumstances, motility plays an important role that allows the bacteria to migrate into a favorable niche and evade intracellular host. Motile bacteria demonstrate its ability to move due to the expression of multiple genes under sophisticated regulatory control, in response to environmental conditions [23,24]. Bacteria can sense a wide range of environmental stimuli such as osmolarity, pH, oxygen tension, temperature, nutrient availability, and consequently adapting their morphology and physiology for survival. Among all these factors, iron depletion causes a reduced production of cellular components, metabolic/enzyme activity and its products [25]. In such condition, pathogens must scavenge available iron sources within the host’s cells to thrive this stressful environment.

Flagella is known to be the functional appendage of bacteria motility. The flagella filaments of *S. maltophilia* are composed of a 38-kDa subunit, SM_FliC_, and appeared to be similar to *Serratia marcescens* (78.6%), *Escherichia coli, Proteus mirabilis, Shigella sonnei* (71.4%), and *Pseudomonas aeruginosa* (57.2%) [26]. Numerous studies have reported the correlation between *S. maltophilia*’s motility and biofilm formation on abiotic surfaces, and its capability of invading host epithelial cells under normal nutritional condition [18,27–30]. Iron depletion in *S. maltophilia* was found to be associated with biofilm formation, extracellular polymeric substances (EPS) production, oxidative stress response, outer membrane proteins (OMPs) regulation, quorum sensing, siderophore production, and expression of iron acquisition systems [31–34]. However, the influence of low iron concentration on motility, especially twitching activity in *S. maltophilia* remains unclear. The motility-associated proteins that are altered during iron depletion also require future investigation. Therefore, this study is aimed at investigating the twitching motility in *S. maltophilia* through *in-silico*, phenotypic and proteomic approaches.

## Materials and methods

### Bacterial strains

A total of 28 clinical isolates (referred to as SM in Table 1) including CS17 (clinical invasive) and CS24 (clinical noninvasive) as reference strains obtained from the laboratory culture collections (Department of Medical Microbiology and Parasitology, Universiti Putra Malaysia, Serdang, Selangor, Malaysia) were used in this study. Five environmental isolates, LMG959, LMG10871, LMG10879, LMG11104, and LMG11108; purchased from Belgian Coordinated Collections of Microorganisms (BCCM) (Laboratorium voor Microbiologie, Universiteit Gent, Belgium) were also studied. The isolates were incubated aerobically for 24 hours at 37°C for clinical and 30°C for environmental isolates [18,21,33].

**Table 1.** *S. maltophiia* strains used in this study and their average zone of twitching under normal and iron-depleted conditions.

				Average zone of twitching in mm (SD)	
*S. maltophilia* isolates	Biological source	Geographical source	Isolates information	Normal	Iron-depleted
***In-silico***					
K279a	Clinical	Bristol, UK	Blood infection		
R551-3	Environmental	Washington, USA	Poplar tree endophyte		
D457	Clinical	Mostoles, Spain	-		
JV3	Environmental	Brazil	Rhizosphere		
**Clinical strains**					
ATCC13637	Pleural fluid	Stafford, England	Oral carcinoma	0	0
CS17	Blood	Malaysia	-	15 (0.58)	20 (1.15)
CS24	Wound swab	Malaysia	-	0	0
SM5	Pus	Malaysia	Surgery	14 (0.58)	20 (2.31)
SM6	Pus	Malaysia	Surgery	24 (2.89)	25 (2.31)
SM7	CSF	Malaysia	Neurosurgery	14 (0.58)	17 (1.73)
SM8	CSF	Malaysia	Neurosurgery	0	0
SM9	Tracheal aspirate	Malaysia	Pediatric	0	0
SM10	Tracheal aspirate	Malaysia	Anesthesiology	0	0
SM11	CSF	Malaysia	Neurosurgery	26 (1.15)	26 (1.15)
SM20	Pus	Malaysia	Pediatric	13 (1.53)	18 (3.46)
SM22	Tracheal aspirate	Malaysia	Pediatric	16 (0.58)	16 (1.63)
SM23	Sputum	Malaysia	Respiratory	14 (0.58)	14 (1.00)
SM24	Blood	Malaysia	Urology and Nephrology	20 (1.53)	25 (1.53)
SM25	Tracheal aspirate	Malaysia	Pediatric	14 (0.58)	17 (1.15)
SM27	Blood	Malaysia	Urology and Nephrology	29 (1.73)	33 (2.08)
SM28	Tracheal aspirate	Malaysia	Pediatric	16 (1.73)	18 (1.73)
SM30	Tracheal aspirate	Malaysia	Anesthesiology	0	0
SM39	Tracheal aspirate	Malaysia	Anesthesiology	34 (1.00)	38 (2.89)
SM40	Blood	Malaysia	Surgery	33 (2.89)	36 (0.58)
SM41	Blood	Malaysia	Pediatric	29 (1.53)	35 (2.52)
SM42	Sputum	Malaysia	Urology and Nephrology	36 (2.31)	40 (4.17)
SM43	Sputum	Malaysia	Medical	0	0
SM44	Pus	Malaysia	Pediatric	22 (1.00)	27 (2.00)
SM45	Tracheal aspirate	Malaysia	Pediatric	45 (0)	50 (2.52)
SM46	Tracheal aspirate	Malaysia	Anesthesiology	0	0
SM47	Urine	Malaysia	Neurosurgery	0	0
SM48	Tracheal aspirate	Malaysia	Medical	34 (3.21)	34 (3.79)
**Environmental strains**					
LMG959	Rice paddy	Japan	-	0	0
LMG10871	Soil	Japan	-	0	0
LMG10879	Rice paddy	Japan	-	23 (0.58)	23 (0)
LMG11104	Roots	Unknown	*Cichorium intybus*, rhizosphere tuberous roots	0	0
LMG11108	Roots	Unknown	Triticum, roots	0	0

0 indicate absence of hazy zone (no twitching motility observed).

### In-silico analysis for targets of twitching motility

The complete genome sequences of four *S. maltophilia* strains, K279a, R551-3, D457 and JV3 (refer to Table 1) were downloaded from the National Center for Biotechnology Information (NCBI, Bethesda, MD, USA) Genbank (www.ncbi.nlm.nih.gov/genome/browse/). The genomes were annotated by Rapid Annotations using Subsystem Technology (RAST) server (http://rast.nmpdr.org/) [35]. Comparative genomics in SEED viewer (Genome Viewer) was used to confirm the identification and conservation of twitching motility genes within *S. maltophilia* genome sequences [36].

### Bacterial culture under iron-depleted and normal conditions

An iron-depleted condition was achieved by adding an iron chelator, 100 µM 2,2ʹ-dipyridyl (DIP) (Sigma Aldrich, Darmstadt, Germany) to LB broth (LB-DIP), while the normal condition was achieved by inoculating the suspension into usual LB broth (without DIP). The tubes were incubated aerobically (37°C for clinical and 30°C for environmental isolates) for 48 hours in an incubator shaker (Model IKA® KS 4000 i control, IKA® Works (Asia) Sdn Bhd, Selangor, Malaysia) at 200 rpm to ensure stationary phase bacterial growth [31,37]. The resulting bacterial growths were adjusted to 0.2 with an Eppendorf BioPhotometer Plus (Hamburg, Germany) at an optical density (OD) of 600 nm.

### Twitching motility assay

*P. aeruginosa* ATCC27853 was used as a positive control as it exhibits twitching motility. Twitching motility was assayed by the modified subsurface agar method previously described [38], with some modifications. A well was made in the center of the twitching agar plate, and 2 µl aliquot of the standardized suspension was inoculated into the well [39]. The zone of twitching was visualized 96 hours after aerobic incubation (37°C for clinical and 30°C for environmental isolates). All assays were carried out at least in triplicate for each of the culture conditions.

### Protein extraction and identification by Isobaric Tags for Relative and Absolute Quantification (ITRAQ)

CS17 and LMG959 strains under both normal and iron-depleted conditions were subjected to ITRAQ analysis. Total protein was extracted from the bacterial supernatant by the trichloroacetic acid (TCA) method. The extracted proteins were quantitated using RC-DC protein assay (Bio-Rad, California, US) as per manufacturer instructions. The ITRAQ assay was outsourced to Proteomic International, Perth, WA, Australia. The ITRAQ assay was performed independently using two biological replicas for each strain at two different conditions in a two 4-plex design. The protein sequences for *S. maltophilia* were obtained from UniProt database using ProteinPilot^TM^ 4.5 software (AB SCIEX, USA). All mass spectrometry-based proteomics data were deposited in the ProteomeXchange Consortium database (http://proteomecentral.proteomexchange.org) via PRoteomics IDEntifications (PRIDE) with the dataset identifier PXD004370.

### Statistical analysis

The twitching motility data were analyzed using IBM SPSS version 24.0. Wilcoxon signed-rank test (related samples, within-group) was used to compare twitching motility under normal versus iron-depleted conditions, respective of clinical and environmental strains. Furthermore, the twitching motility between clinical and environmental isolates under two growth conditions was analyzed using the Mann-Whitney test (unrelated samples, different group). Lastly, the differences in the strains that showed twitching positive and negative strains were analyzed. The p-values <0.05 is considered statistically significant for all the analysis. For proteomic analysis, an average protein ratio and p-values, which indicate significant differential expression was calculated by ProteinPilot^TM^ 4.5 software (AB SCIEX, USA).

## Results

### Targets of twitching motility in S. maltophilia

Targeted *in-silico* analysis of the four complete genomes of *S. maltophilia* (see Table 1), revealed the presence of shared type IV pilus subsystem across all the strains. This subsystem is categorized under “membrane transport” and subcategorized under “protein and nucleoprotein secretion system, type IV”. The 21 targets and functional roles obtained from RAST server are listed in Table 2.

**Table 2.** Functional roles of type IV pilus associated with twitching motility and their abbreviations obtained from RAST server.

Targets^a^	Functional Role	Locus tag^b^
DQS	3-dehydroquinate synthase	SMLT_RS17230
pilD	Leader peptidase (Prepilin peptidase)	SMLT_RS17910
MTT	Multimodular transpeptidase-transglycosylase	SMLT_RS17525
pilDm	N-methyltransferase	SMLT_RS17910
pilT	Twitching motility protein PilT	SMLT_RS05270
pilSe	Two-component sensor PilS	SMLT_RS17880
pilR	Type IV fimbriae expression regulatory protein PilR	SMLT_RS17885
pilC	Type IV fimbrial assembly protein PilC	SMLT_RS17905
pilB	Type IV fimbrial assembly, ATPase PilB	SMLT_RS17890
fimT	Type IV fimbrial biogenesis protein FimT	SMLT_RS07795
pilV	Type IV fimbrial biogenesis protein PilV	SMLT_RS07795
pilW	Type IV fimbrial biogenesis protein PilW	SMLT_RS07800
pilX	Type IV fimbrial biogenesis protein PilX	SMLT_RS07810
pilY1	Type IV fimbrial biogenesis protein PilY1	SMLT_RS07815
pilA	Type IV pilin PilA	SMLT_RS17895
pilE	Type IV pilus biogenesis protein PilE	SMLT_RS07820
pilM	Type IV pilus biogenesis protein PilM	SMLT_RS18210
pilN	Type IV pilus biogenesis protein PilN	SMLT_RS18205
pilO	Type IV pilus biogenesis protein PilO	SMLT_RS18200
pilP	Type IV pilus biogenesis protein PilP	SMLT_RS18200
pilQ	Type IV pilus biogenesis protein PilQ	SMLT_RS18195

^a^The abbreviations of the targets and name of the functional roles are derived from RAST server; ^b^Corresponding locus tag respective to *S. maltophilia K279a* from GenBank, NCBI.

### Twitching motility in S. maltophilia

**Figure 1.**
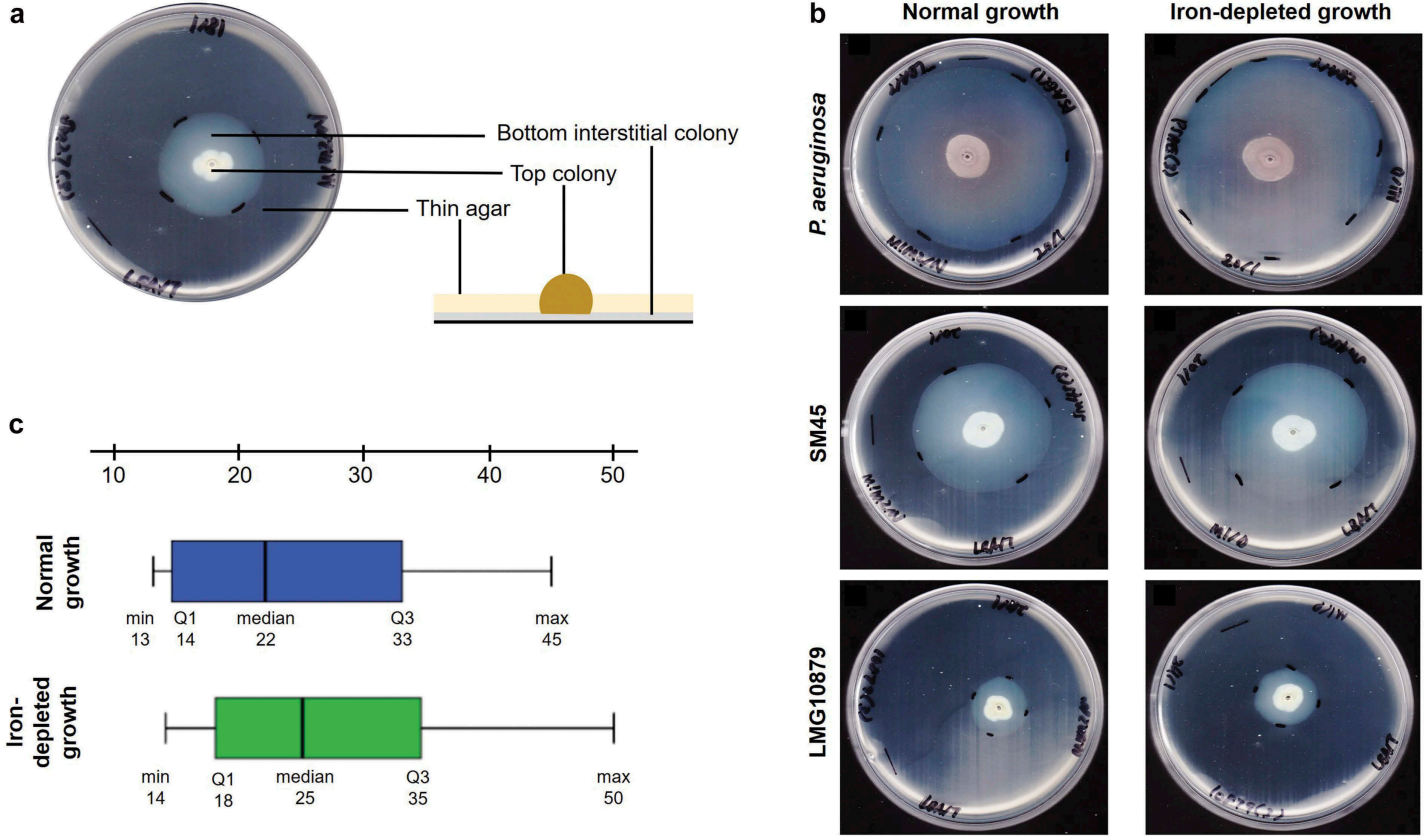
(a) Typical subsurface twitching motility agar interpretation. A colony on the surface of agar around the inoculation point (top colony) and a visible halo or hazy zone of bacteria that have twitched across the plate between the bottom of the agar and the Petri plate (interstitial colony). (b) A representative of the zone of twitching motility on twitching agar plates on normal and iron-depleted conditions. *P. aeruginosa* ATCC27853 (positive control), SM45 (clinical isolate) and LMG10879 (environmental isolate). The plates were flooded with TM developer solution. The zone of twitching motility was marked, and 1 cm line was drawn onto the plates. (c) Box-and-whisker plot for clinical isolates under normal and iron-depleted conditions. The twitching zone under normal condition (min: 13 mm, median: 22 mm, max: 45 mm), while iron-depleted condition (min:14 mm, median: 25 mm, max: 50 mm).

The zone of twitching measured after 96 hours is tabulated in Table 1. Typical subsurface twitching motility agar interpretation is shown in Figure 1(a). The zone of twitching motility on twitching agar plates on normal and iron-depleted conditions for *P. aeruginosa*, SM45 and LMG10879 are shown in Figure 1(b). *P. aeruginosa* exhibited an average of 67 mm and 71 mm in normal and iron-depleted conditions respectively. Among the 33 *S. maltophilia* tested, 19 clinical isolates and one environmental isolate exhibited twitching motility in both conditions. The clinical isolates produced a zone of twitching with a mean of 22.00 mm under normal and 25.00 mm under iron-depleted conditions. The statistical analysis showed p < 0.05 (0.002), inferring significant difference between a zone of twitching under normal and iron-depleted conditions among 28 clinical strains tested. Figure 1(c) shows box-and-whisker plot (boxplot) for clinical isolates under normal and iron-depleted conditions.

On the other hand, the only environmental strain, LMG10879, although twitched in the normal condition did not show any increase in zone size under iron-depleted condition (23.00 mm in both conditions), thereby no significant difference was found (p = 1.000). Furthermore, there was no significant difference in twitching motility between clinical and environmental isolates under normal and iron-depleted conditions, with p = 0.104 and p = 0.083, respectively. Those strains (n = 20) that exhibited positive twitching activity, there was no significant difference for all isolates under normal and iron-depleted conditions, with p = 1.000 and p = 0.900 respectively. While, strains (n = 13) that exhibited negative twitching activity, there was no significant difference for all isolates under normal and iron-depleted conditions, with p = 1.000 and p = 1.000 respectively.

### Motility-associated proteins of S. maltophilia under iron-depleted

A total of 687 differentially expressed proteins were detected, of which 151 were commonly found to be present in both replicates. Among the 687 proteins detected, a total of 122 proteins (58 up-regulated and 19 down-regulated in CS17 and 36 up-regulated and seven down-regulated in LMG959) were seen with altered expression in response to iron-depleted condition (data not shown). The motility-associated proteins that were detected under normal versus iron-depleted conditions for both CS17 and LMG959 are shown in Table 3. The ITRAQ analysis showed that, only targets for CS17 was found significantly different between normal and iron-depleted conditions, including: (1) Flagellar biosynthesis protein FliC, *S. maltophilia* EPM1, 20.14-fold change, p = 0.0065; (2) Flagellar biosynthesis protein FliC, *S. maltophilia* D457, 1.54-fold change, p = 0.0046; (3) Fimbrial protein, *S. maltophilia* EPM1, 0.70-fold change, p = 0.0147; and (4) twitching motility protein PilT, *S. maltophilia* 5BA-I-2, 99.08-fold change, p = 0.0156.

**Table 3.** Differentially expressed motility-associated proteins identified via ITRAQ assay for CS17 and LMG959 isolates under iron-depleted condition.

Protein name	Accession number^a^	Percentage coverage^b^	Fold change^c^	p-value^d^
**CS17**				
Flagellar biosynthesis protein FliC	A0A0X3QSM8	73.5	**20.14**	**0.0065**
Flagellar biosynthesis protein FliC	A0A2Y9U6E5	75.0	**1.54**	**0.0046**
Pili assembly chaperone: bacterial pili assembly chaperone	A0A0U5I3M8	27.7	3.66	0.5431
Fimbrial protein	A0A0U5I9J4	33.5	**0.70**	**0.0147**
Twitching motility protein PilT	A0A501PPD4	13.6	**99.08**	**0.0156**
**LMG959**				
Flagellar biosynthesis protein FliC	A0A0X3QSM8	75.3	17.70	0.2522
Flagellin	A0A2W6J2T0	50.0	1.43	0.6261
Fimbrial protein	A0A064BLK2	7.4	0.64	0.2778
Flagellar biosynthesis protein FliC	A0A0M0NGJ6	33.7	2.19	0.9019
Pili chaperone protein	A0A2T1I8L3	19.0	18.37	0.1309

^a^UniProt protein accession number, ^b^ Percentage of identified peptides over the whole protein sequence, ^c^ Fold change comparing protein level for the strain growing under iron-depleted versus normal iron conditions, ^d^ the fold changes in **bold** are consider statistically significant, set at (p < 0.05).

## Discussion

Our *in-silico* analysis revealed that adhesion and motility factors such as pili and fimbriae were found in all *S. maltophilia* genomes (see Table 2). A previous study reported that, *S. maltophilia* isolates K279a and SKK35 (clinical strains), R551-3 (environmental strain), SKA14 (seawater strain), and RA8 (wastewater strain) were found to harbor genes involved in pili and fimbriae formation, including: (1) Twitching motility proteins (*pilU, pilT, pilE, pilI, pilJ, pilH, pilG, cheAW*); (2) Fimbrial proteins (*smf1*); (3) Pilus assembly proteins (*pilZ, pilW, pilV, pilX, pilY, pilE, pilF, pilQ, pilP, pilO, pilN, pilM*); and (4) Twitching motility proteins, pilus assembly proteins (*pilS*) [40]. Notably, both environmental and clinical strains of *S. maltophilia* have been found to contain genes encoding for twitching motility. In such circumstances, *S. maltophilia* shows the dual nature (i.e., commensal and true pathogen) [34], and it is utmost essential to treat it as health hazard even when isolated from environment [41].

*P. aeruginosa* exhibited active twitching motility at the agar-plate interface, with radial rates of expansion approaching 1 mm/h, resulting in large but fine twitching zones approaching 20 to 30 mm in diameter after overnight growth [42]. In our study, nearly similar zone sizes were found, as, within four days of incubation, an average of 67 mm and 71 mm in normal and iron-depleted conditions respectively. These results were in accordance with several earlier studies which showed enhanced twitching motility for *P. aeruginosa* under reduced iron concentration [43–45]. In the present study, a variance of twitching pattern was observed, where only 60.6% exhibited the twitching motility. This variability could be attributed to the source of the isolates, and a larger zone of twitching was observed for isolates obtained from blood [28]. In our study, five clinical isolates from blood exhibited larger zone of twitching under iron-depleted compared to normal conditions (20.00 mm for CS17, 25.00 mm for SM24, 33.00 mm for SM27, 36.00 mm for SM40, 35.00 mm for SM41) (see Table 1), but not statistically significant (p = 0.136). In such circumstances, high disease severity and mortality were associated with hospital-acquired *S. maltophilia* bacteremia in comparison with community [46–50].

In contrast, some studies also reported the absence of twitching motility among *S. maltophilia* [39,43]. Aforementioned, only 19 clinical isolates formed a hazy zone with different diameter of the zone, suggestive that not all strains are capable of twitching motility. Regarding environmental isolates, a study reported that LMG959 showed a zone of twitching under normal growth condition [27], but we did not observe in our study. Instead, LMG10879 exhibited twitching with an average zone of 23.00 mm in both normal and iron-depleted conditions. Environmental strains exhibit twitching activity and may not necessarily iron-dependent, thus deemed further investigation to identify other factors.

**Figure 2.**
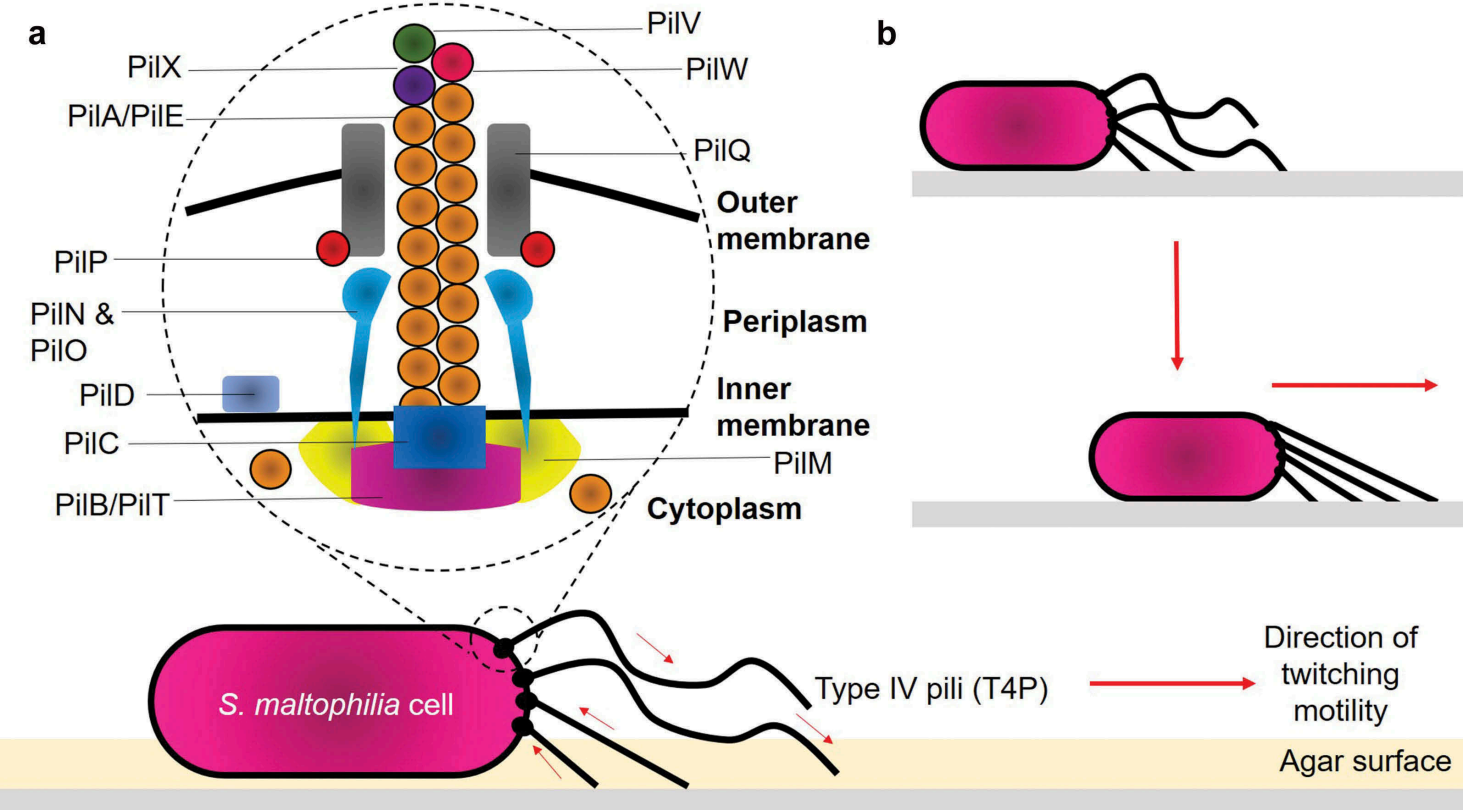
Structure of type IV pili and mechanisms of bacterial twitching motility. (a) Structurally, T4P is composed of: (1) Major pilin PilA/PilE; (2) Secretin PilQ; (3) Alignment proteins PilM, PilN, PilO, PilP; (4) Retraction ATPase PilT; (5) Assembly ATPase PilB; and (6) Minor pilins PilX, PilW, PilV. (b) Pilus retraction results in the forward movement of the cell across the surface. Arrows indicate the direction of pilus retraction/extension.

The ITRAQ analysis showed that, CS17 was found significantly to fimbrial-related protein. (A0A0U5I9J4, 0.70- fold change, p = 0.0147). The expression of pili/fimbriae is crucial for adhesion and biofilm formation [26]. Particularly, the Smf-1 fimbrial operon in *S. maltophilia* mediates adherence at early stages of biofilm formation [51]. Smf-1 is a peritrichous semiflexible fimbria of 5 to 7 nm under electron microscopy and only formed at 37°C, suggesting the reason why there was no significant change-fold observed in LMG959, as this strain was grown at 30°C. The flagellar biosynthesis protein FliC (A0A0X3QSM8, 20.14- fold change, p = 0.0065; A0A2Y9U6E5, 1.54-fold change, p = 0.0046) is relatively important, as it mediates the attachment of bacteria to epithelial cells, and initiate host’s infection process [52]. Interestingly, the twitching motility protein PilT (A0A501PPD4, p = 0.0156) was found the highest (99.08-fold change) in *S. maltophilia*. Twitching motility was proposed to be mediated by type IV pili (T4P) located at one or both poles of the cells. Structurally, the pili are 5 to 7 nm in diameter and composed of small protein subunit called PilA (also known as pilin). T4P is capable of binding to a variety of surfaces such as inert surfaces, bacterial or eukaryotic cells, thereby exhibiting contact and promote colonization via a specific mechanism [42,53]. A model for *S. maltophilia* type IV pilus can reasonably be proposed based on RAST server and previous other studies on *P. aeruginosa* [54–56] as shown in Figure 2(a). It was elucidated that functional retractile pili are the mechanical basis for twitching motility. The removal of PilA from the base of assembled pilus fiber, followed by shortening of the pilus, pulls the cell closer to the site of attachment (Figure 2(b)) [54,57–59].

Twitching motility may facilitate the bacteria to spread in the infected tissue, particularly in patients with the iron deficit, and promote tissue colonization to initiate its pathogenesis. Hence, further studies are warranted to determine why not all *S. maltophilia* strains have the ability to twitch (i.e., environmental isolates) and also to understand the biology behind increased twitching during reduced iron availability in an *in-vivo* model.

## Data Availability

The datasets generated and/or analysed during the current study are available in the ProteomeXchange Consortium database (http://proteomecentral.proteomexchange.org) via PRoteomics IDEntifications (PRIDE) with the dataset identifier PXD004370.
